# Studies About the Effect of Halogenated Solvents on the Fluorescence Properties of 9-Aryl-Substituted Isoquinolinium Derivatives – A Case Study

**DOI:** 10.1007/s10895-024-03691-z

**Published:** 2024-04-10

**Authors:** Philipp Groß, Heiko Ihmels

**Affiliations:** Department of Chemistry and Biology, Center of Micro- and Nanochemistry and (Bio)Technology (Cµ), University of Siegen, Adolf-Reichwein-Str. 2, 57068 Siegen, Germany

**Keywords:** Berberine, Cationic dye, Halogen bond, Solvatochromism

## Abstract

It was demonstrated that 9-aryl-substituted isoquinolinium derivatives have significantly increased fluorescence quantum yields in halogenated solvents, mostly pronounced in chloroalkanes, which appears to be specific for this type of solvents. Further analysis with selected halogenated solvents revealed that the type and number of halogen substituents and the dielectric constant of the solvent have a distinct impact on the emission quantum yield. The solvent effect is explained by a solvation of the charge shift (CS) state by attractive halogen–π interactions (halogen bond), which impedes the torsional relaxation of the excited state.

**Supplementary Information:**

The online version contains supplementary material available at 10.1007/s10895-024-03691-z.

## Introduction

**Fig. 1:**
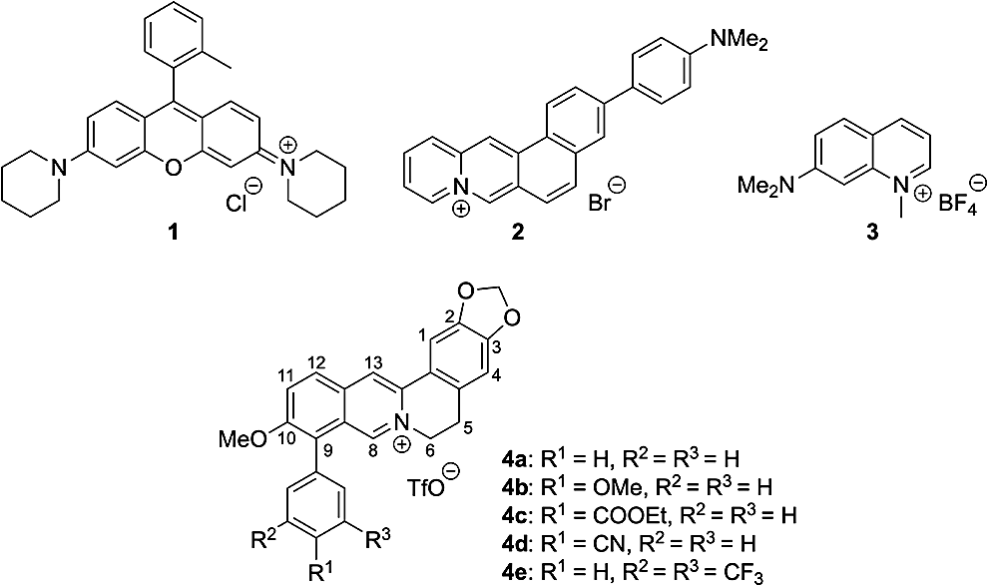
Structures of rosamine (**1**), naphtho[1,2-*b*]quinolizinium **2**, 1-methylquinolinium **3** and 9-aryl-substituted berberine derivatives **4a**–**e** (TfO = Trifluoromethylsulfonate)

The absorption and emission properties of organic dyes in solution often depend on the employed solvent [1, 2]. This well-known medium effect is mainly caused by the individual solvent parameters, which may influence the stabilization or destabilization of the solute molecule in the ground and excited state and, as a consequence, also affect the photophysical properties [1]. The extent of the solvent effects depends on unspecific and specific interactions between the solvent and the respective solute, which includes, for example, dipole-dipole interactions, hydrogen bonding, or electrostatic interactions [3]. Because of this, solvent properties, such as polarity, polarizability, hydrogen bond accepting (HBA) or hydrogen bond donating (HBD) properties, etc. may be assessed qualitatively and quantitatively with the help of reporter molecules, namely solvatochromic probes, whose photophysical properties correlate well with particular solvent properties [4–6]. In this context, it has been observed frequently that some compounds show special, even extraordinary properties in halogenated alkane solvents [7–9]. And these effects have been explained by the high electronegativity of the halogen substituents and the resulting regions with high electron density of the solvent molecule, which in turn enable attractive Coulomb interactions with the positively polarized regions of the solute molecule [10, 11]. However, the electrostatic potential surface of the halogen atom, especially of the heavy ones, also has a region of low electron density, which may result in attractive interactions with electron-rich sites of the solute [11]. The general capability of halogen substituents to associate with electron-rich regions of other funtionalities was understood only recently, and this so-called halogen bond has developed into an important element in many areas of chemistry, such as crystal engineering and medicinal chemistry [10–16]. For example, the combination of halogen bonds and hydrogen bonds was successfully applied to increase the stability and activity of enzymes [10]. Furthermore, the interaction between a halogenated solvent and a solute were already used for sensing applications [17–19]. However, the effect of halogenated solvents on the emission properties of the solute molecule was only investigated in sufficient detail in a few cases [8, 20–22]. And it has been reported occasionally that cationic dye molecules, such as, for example, rosamine (**1**) [23], naphtho[1,2-*b*]quinolizinium **2** [24], or 7-(dimethylamino)-1-methylquinolinium (**3**) (Fig. 1) [25], display enhanced fluorescence quantum yields in dichloromethane and chloroform solution, which indicates a special interaction of the solvent molecules with the dye in the excited state in these cases [23–27]. But this effect has not been further investigated with a focus on specific solute-solvent interactions, and correlations between the solvent parameters of the halogenated solvents and the fluorescence quantum yields of cationic dye molecules have not been systematically assessed, so far.

Recently, we have discovered that the 9-arylsubstituted berberine-type derivatives **4a**–**e**, also referred to as annelated isoquinolinium salts, have very low intrinsic fluorescence quantum yields in non-halogenated solvents, namely in MeOH, DMSO and MeCN (*Φ*_fl_ ≤ 0.02) [28, 29]. This weak emission was explained by a radiationless deactivation of the excited state by a twisted intramolecular charge transfer (TICT) process, which involves torsional relaxation about the biaryl axis at position C9 [29]. However, orienting experiments have shown that the fluorescence quantum yields of these compounds are significantly enhanced in halogenated solvents. Considering the yet not fully understood effect of halogenated solvents on the emission properties of cationic dyes and the potential use of this effect in fluorescence-based sensing, we further investigated the fluorescence quantum yields of these derivatives in selected halogen-containing solvents to examine whether this is a general effect of halogenated solvents on this class of compounds.

## Methods

The absorption spectra were recorded on a Varian Cary 100 Bio absorption spectrometer with Hellma quartz glass cuvettes 115 F-QS (layer thickness *d* = 10 mm). The emission spectra were measured on a Varian Cary Eclipse fluorescence spectrometer with Hellma quartz glass cuvettes 115 F-QS (layer thickness *d* = 10 mm). If not stated otherwise, all absorption and emission measurements were recorded at *T* = 20 °C as adjusted with a thermostat. The sample solutions were mixed with a reaction vessel shaker Top-Mix 11,118 (Fisher Bioblock Scientific). The elemental analysis data were determined in-house with a HEKAtech EUROEA combustion analyzer.

## Results

**Fig. 2:**
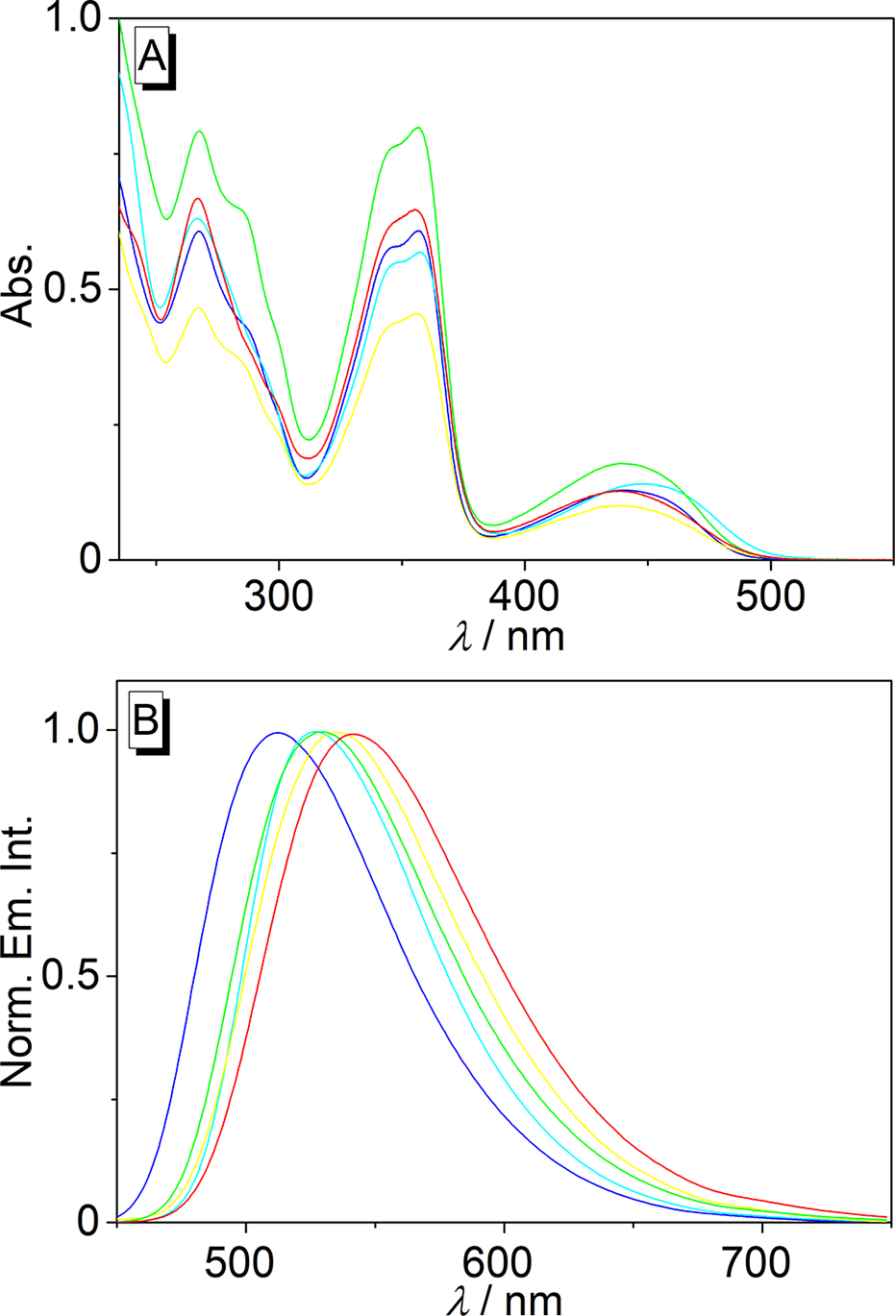
Absorption (**A**) and emission spectra (**B**) of **4a** (blue), **4b** (cyan), **4c** (green), **4d** (yellow), and **4e** (red) in CH_2_Cl_2_ (*c* = 20 µM, *λ*_ex_ = 421 nm)

In CH_2_Cl_2_ solution the compounds **4a**–**e** absorb essentially in the same range as in MeCN (Table 1), MeOH and DMSO (cf. Supporting Information Figure S1 and S2) [29]. Hence, derivative **4b** showed a broad, red-shifted absorption maximum at 452 nm, whereas for derivatives **4a**,**c**,**d**,**e** a similar structured absorption maximum around 440 nm was observed (Fig. 2). All compounds showed blue-shifted, more intense, but unstructured absorption bands with maxima at 356 nm and between 230 and 270 nm. The high-energy bands showed additional, red-shifted shoulders, mostly pronounced for **4c** (Fig. 2A; Table 1) [29].

**Table 1:** Absorption and emission properties of berberine derivatives **4a**–**e** in CH_2_Cl_2_ and MeCN

	CH_2_Cl_2_			MeCN		
	*λ*_abs_ / nm (log ε)	*λ*_fl_ / nm	*Φ*_fl_^a^	*λ*_abs_ / nm (log ε)	*λ*_fl_ / nm	*Φ*_fl_^a^
4a	441 (3.81) 357 (4.48) 287 (4.33)^b^ 267 (4.48)	512	0.19	425 (3.78) 348 (4.41) 277 (4.34)^b^ 263 (4.41)	565	< 0.01
4b	449 (3.85) 357 (4.45) 293 (4.32)^b^ 267 (4.50) 232 (4.72)	526	0.28	430 (3.85) 349 (4.42) 277 (4.37)^b^ 263 (4.44) 232 (4.57)	538	0.01
4c	440 (3.95) 357 (4.60) 286 (4.51)^b^ 267 (4.60)	529	0.17	424 (3.88) 348 (4.50) 278 (4.47)^b^ 263 (4.52)	572	< 0.01
4d	439 (3.70) 356 (4.36) 284 (4.27)^b^ 267 (4.37)	535	0.06	424 (3.86) 347 (4.47) 279 (4.42)^b^ 263 (4.49)	573^c^	< 0.01
4e	438 (3.80) 355 (4.51) 287 (4.29)^b^ 267 (4.52)	542	0.08	422 (3.83) 347 (4.48) 278 (4.38)^b^ 263 (4.51)	570	< 0.01

^a^ Fluorescence quantum yield relative to coumarin 153 in EtOH (*Φ*_fl_ = 0.38) Ref [30]; estimated error: ±10% of the given data. ^b^ Maximum refers to a shoulder in the absorption. ^c^ The emission maximum has a significant margin of error because of an exceptionally low emission intensity

**Fig. 3:**
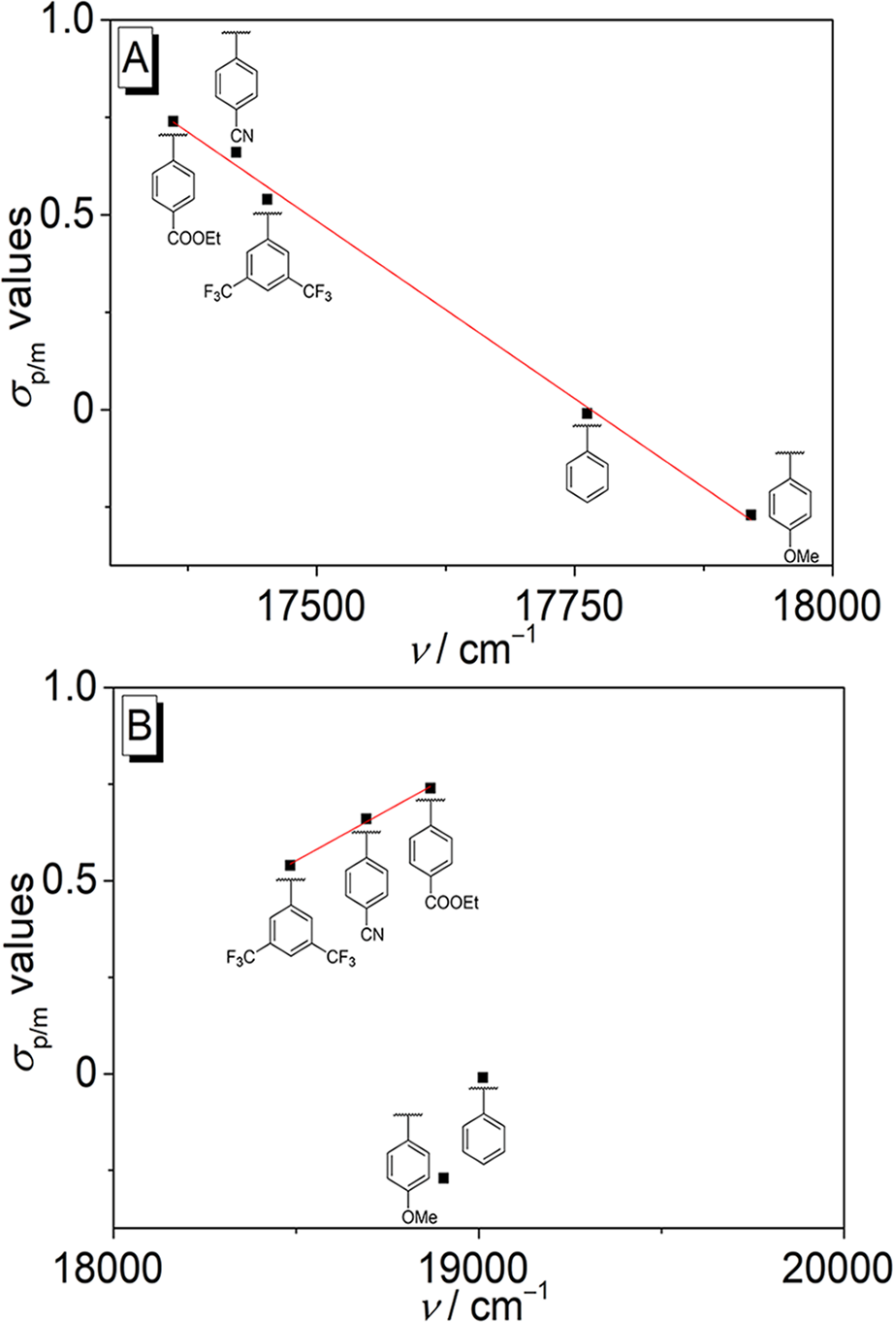
Plot of the Hammett constants (*σ*_*p*/*m*_ values) versus emission maxima (in wavenumbers) of derivatives **4a**–**e** in MeCN (**A**) or CH_2_Cl_2_ (**B**)

In contrast, the emission spectra of the derivatives **4a**–**e** differed significantly depending on their substitution pattern. Namely, the fluorescence spectrum of the 9-phenyl-substituted derivative **4a** exhibited an emission maximum at 512 nm, whereas the emission bands of the substituted derivatives were significantly red shifted with increasing electron-acceptor properties of the substituents. Accordingly, the methoxy-substituted derivative **4b** had an emission maximum at 526 nm, and the acceptor-substituted compounds **4c**, **4d**, and **4e** exhibited red-shifted emission maxima at 529 nm, 535 nm, and 542 nm, respectively (Fig. 2B). For further evaluation of the dependency of the emission energies of the 9-phenyl-substituted isoquinolinium derivatives on the substitution pattern, the Hammett constants [31] of the functionalities at the phenyl substituent were plotted versus the emission energies in wavenumbers in CH_2_Cl_2_ or in MeCN (Fig. 3). The plot of the data in MeCN as solvent revealed a good correlation (*R*^2^ = 0.99) of the *σ* values of the phenyl-substituted derivatives with the fluorescence maxima, specifically the emission energy decreases almost linearly with increasing *σ* value (Fig. 3A). In contrast, no general correlation (*R*^2^ = 0.99) between the Hammett constants and the emission energy of the compounds **4a**–**e** was observed in CH_2_Cl_2_ solution. But at least the electron acceptor-substituted derivatives **4c**–**e** showed a linear increase of emission energy with increasing *σ* values (Fig. 3B), albeit the changes of emission energy are relatively small in this group.

It has been shown already that the very low fluorescence quantum yields of the derivatives **4a**–**e** in halogen-free solvents (*Φ*_fl_ < 0.02) are caused by torsional relaxation about the biaryl axis in the excited state, which provides routes for radiationless deactivation [29]. In sharp contrast, however, considerably higher quantum yields were detected for compounds **4a**–**e** in CH_2_Cl_2_ (*Φ*_fl_ = 0.06–0.28) as compared with the ones observed in halogen-free solvents. More specifically, the derivatives **4d** and **4e** displayed the lowest fluorescence quantum yields in CH_2_Cl_2_ (*Φ*_fl_ = 0.06–0.08), whereas the derivatives **4a** and **4c** had values of *Φ*_fl_ = 0.17–0.19. The highest fluorescence quantum yield was determined for the methoxy-substituted compound **4b** with *Φ*_fl_ = 0.28 in CH_2_Cl_2_ (Table 1).

**Fig. 4:**
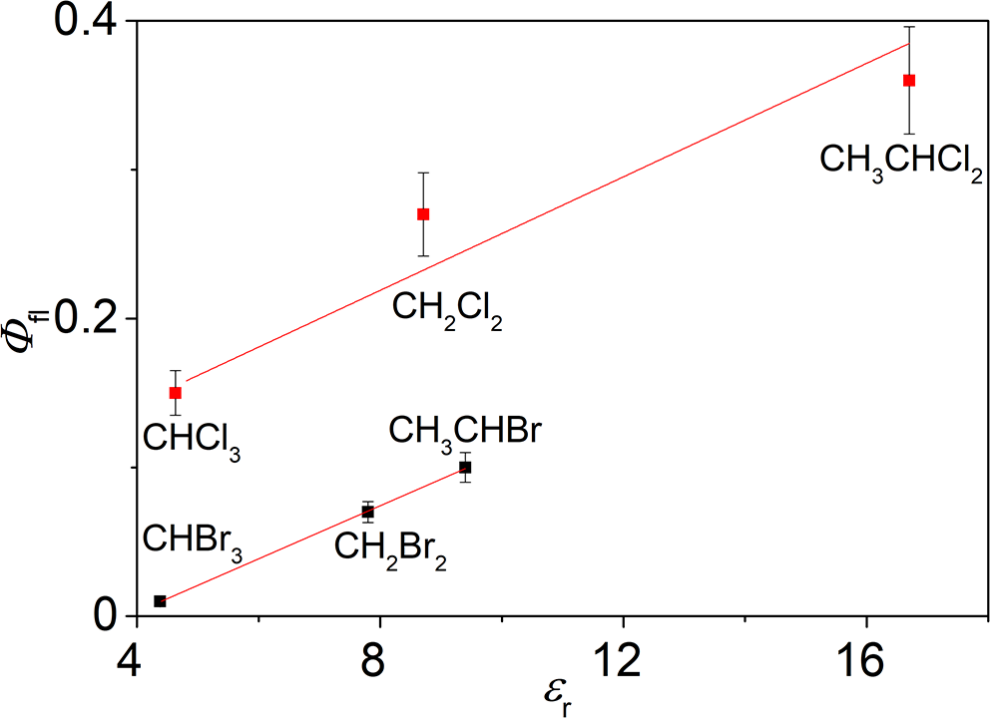
Plot of the fluorescence quantum yields (*Φ*_fl_) of **4b** in chloroalkanes (red) and bromoalkanes (black) versus the dielectric constants (*ε*_r_) of the solvents

To further investigate the fluorescence properties of 9-arylisoquinolinium ions in representative halogenated solvents, the 4-methoxy-substituted derivative **4b**, which showed the highest fluorescence quantum yield in CH_2_Cl_2_, was investigated exemplarily in a series of selected halogenated solvents (Table 2, cf. Supporting Information, Figure S3). These measurements revealed a distinct influence of the dielectric constant as well as the type and number of halogen atoms on the fluorescence quantum yield of **4b**. In general, the fluorescence quantum yields are lower in brominated solvents (*Φ*_fl_ = 0.01–0.10) than in the chlorinated ones (*Φ*_fl_ = 0.15–0.36). Specifically, **4b** exhibited the highest fluorescence quantum yield in 1,1-dichloroethane (*Φ*_fl_ = 0.36, *ε* = 16.7) and the lowest quantum yield in bromoform (*Φ*_fl_ = 0.01, *ε* = 4.38). Furthermore, the bromoalkanes as well as the chloroalkanes showed correlations (*R*^2^ = 0.96, 0.99) in a plot of the fluorescence quantum yields of **4b** versus the dielectric constants of the respective solvents (Fig. 4). Noteworthy, a plot of other solvent parameters versus the fluorescence quantum yield gave no obvious correlation. And in some cases, the required solvent parameters for the solvents used in this study were not available in the literature. Noteworthy, in non-halogenated solvents a meaningful correlation between the dielectric constants and the fluorescence quantum yields of **4b** could not be obtained because of the very low emission intensity (*Φ*_fl_ ≤ 0.01) in these media.

**Table 2:** Fluorescence properties of 9-(4-methoxyphenyl)isoquinolinium **4b** in different halogenated solvents

Solvent	*λ*_fl_ / nm		*Φ*_fl_^a^		*ε*_r_^b^
	TfO^−^	Br^−^	TfO^−^	Br^−^	
CH_3_CHCl_2_	528	–^c^	0.36	–^c^	16.7
CH_2_Cl_2_	527	526	0.28	0.21	8.71
CHCl_3_	531	529	0.15	0.02	4.63
CH_3_CH_2_Br	527	527	0.10	0.09	9.40
CH_2_Br_2_	531	–^c^	0.07	–^c^	7.80
CHBr_3_	533	531	0.01	0.01	4.38

^a^ Fluorescence quantum yield relative to coumarin 153 in EtOH (*Φ*_fl_ = 0.38) Ref [30]; estimated error: ±10% of the given values. ^b^ Relative permittivity (“dielectric constant”); Ref [32]. ^c^ Not soluble in the respective solvent

To investigate the influence of the counter anion on the fluorescence quantum yields, the derivative **4b** was transformed to the corresponding bromide salt **4b**^**Br**^ by ion metathesis (cf. Supporting Information), and the absorption and emission properties were determined in halogenated solvents. The bromide salt was not sufficiently soluble in 1,1-dichloroethane and CH_2_Br_2_. In general, the absorption bands of derivatives **4b** and **4b**^**Br**^ are the same in the tested solvents. Likewise, the shifts of the emission bands did not change significantly. And in bromoethane and CHBr_3_, in which the emission intensity of **4b** is already relatively weak, similarly low fluorescence quantum yields were observed with **4b**^**Br**^ (*Φ*_fl_ = 0.10 and 0.01, Table 2). In contrast, however, the exchange of TfO^−^ with Br^−^ led to a decrease of the fluorescence quantum yields in chlorinated solvents (Table 2, cf. SI Figure S4). Specifically, the fluorescence quantum yields decreased by 86% in CHCl_3_ (*Φ*_fl,**1b**_ = 0.15, *Φ*_fl,**1bBr**_ = 0.02) and by 25% in CH_2_Cl_2_ (*Φ*_fl,**1b**_ = 0.28, *Φ*_fl,**1bBr**_ = 0.21).

## Discussion

**Scheme 1:**
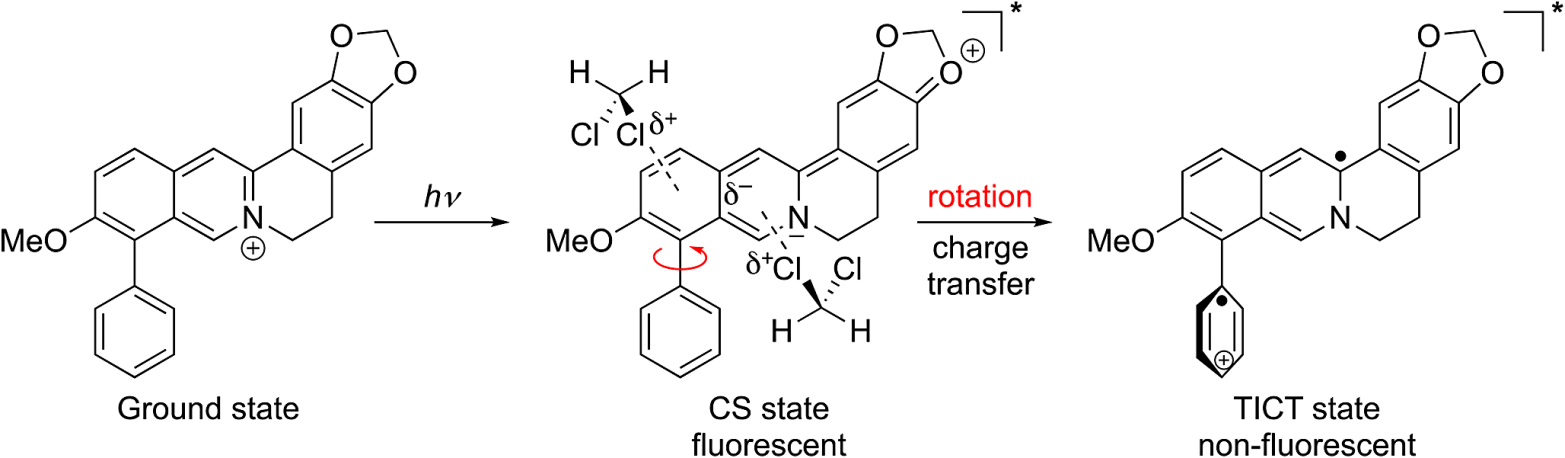
Schematic presentation of the formation of the charge shift (CS) state and twisted internal charge transfer (TICT) state upon excitation of 9-aryl-substituted berberine-type isoquinolinium and solvation of the CS state by dichloromethane

It has already been shown that the introduction of an aryl-substituent in 9-position quenches the intrinsic fluorescence of the fluorophore by a non-emissive TICT process, and that this deactivation route can be suppressed when the rotation about the biaryl axis is hindered (Scheme 1), for example by the increase of the viscosity of the solvent or by the association within a sterically constrained binding site [29]. Therefore, it may be assumed that the increase in emission intensity of compound **4a**–**e** in halogenated solvents compared with the intensity observed in halogen-free solvents is caused by specific solute-solvent interactions, presumably by a halogen bond [11], which influence this biaryl rotation. And as only an insignificant effect of the halogenated solvents on the absorption properties was observed (Fig. 1; Table 1), the relevant interactions must be identified in the excited state. In the case of the parent berberine, it has been shown that upon excitation a significant charge redistribution, in the extreme case a charge shift (CS), is induced from the benzodioxole fragment to the isoquinolinium unit (Scheme 1) [33]. And essentially the same shift of electron density is induced by excitation of the derivative **4b**, as shown by time-dependent density functional theory (TD-DFT) calculations (cf. Supporting Information, Figure S5) [33]. The proposed formation of the CS state is also supported by the reasonable correlation of emission energies of **4a**–**e** with the Hammett parameters in acetonitrile solution. Specifically, the red shift of the emission maximum with increasing acceptor property of the phenyl substituent in **4c**–**e** shows that the latter enables a more pronounced donor-acceptor interplay with the electron-rich enamine/enolether-type structure in the CS state. If the electron distribution of the isoquinolinium ion was maintained upon excitation a more pronounced red shift would have been expected with donor-substituted derivatives **4a**,**b**.

In haloalkanes the solvent effect is obviously different. It has been shown already for the parent berberine that the rates of intersystem crossing (ISC) increase in several non-polar solvents like dichloromethane [34, 35]. Therefore, lower fluorescence quantum yields in halogenated solvents than in non-halogenated ones should have been expected also for derivatives **4a**–**e**. In contrast, even a significant increase in emission intensity was observed. Therefore, the solvent effect on derivatives **4a**–**e** is likely caused by the additional aryl substituent at the berberine core. Considering the pronounced positively polarized regions at the halogen substituent, attractive Coulomb interactions may result between the electron-rich isoquinoline unit of the berberines **4a**–**e** in the excited state and the electron-deficient part of the halogen atoms of the solvent, the so-called *σ*-hole [11, 33]. It may be proposed that these halogen–π interactions lead to the formation of a more compact solvent shell which includes the aryl substituent in 9-position, such that the energy barrier for the rotation about the biaryl axis is increased and torsional relaxation is hindered (Scheme 1) [10–12, 33]. This particular solvation mode may also explain the lack of correlation of the emission energy of **4a**–**e** with the Hammett constants of the phenyl substituents. As the electrons of the quinoline unit in the CS state participate in the attractive interactions with the solvent molecules, a significant donor-acceptor interplay with the phenyl ring (see above) cannot be established. Finally, this model is consistent with the correlation of the emission quantum yield of **4b** with the dielectric constants of the halogenated solvents. Taking the *ε*_r_ values as rough measure for solvent polarity, the higher quantum yields are explained by the better stabilization of the complex between the polarized solute and solvent by the more polar medium.

It should be noted that the simplified model described above does not fully apply to brominated solvents. In the latter case, the situation is obviously interfered with by additional deactivation pathways because the emission quantum yields are relatively low (Table 2). This effect may indicate that – other than in chlorinated solvents – the solvent-solute interaction is much weaker and that the resulting solvent shell does still allow for significant deactivation of the excited molecule by rotation about the biaryl axis [36]. Moreover, the fluorescence quenching may also be caused by increased intersystem crossing rates induced by the heavy atom effect of the bromine atoms [37].

The counter ion metathesis of **4b** to **4b**^**Br**^ led to a decrease of the fluorescence quantum yield. This effect may be caused by a better solvation of the bromide counter ion, which promotes the ion migration during the excited state charge-shift process and supports the fluorescence quenching [38]. Accordingly, this effect is most pronounced in chloroform that has been shown to form highly defined solvent shells around halide ions, thus enhancing their mobility in solution [39]. At the same time, the counter ion exchange did not induce changes of the emission energy, which indicates that the ion metathesis influences the dynamics in the excited state, but not the energy levels of the CS and TICT state.

## Conclusion

In summary, it was shown that isoquinolinium **4a**–**e** have significantly increased fluorescence quantum yields in halogenated solvents, mostly pronounced in chloroalkanes, which appears to be specific for this this type of solvents. In a case study, a qualitative analysis revealed that the type of halogen substituents and the dielectric constant of the solvent have a distinct impact on the emission quantum yield. The solvent effect is explained by a solvation of the CS state by attractive halogen–π interactions, which impedes the torsional relaxation of the excited state. Although these results cannot be generalized, they are in agreement with the observation that such solvent effects of chloroalkanes have been mainly observed with cationic dyes. As the latter commonly tend to form CS states upon excitation, it may be proposed that a similar type of solvation as with dyes **4a**–**e** takes place. Certainly, more detailed, systematic studies are required to clarify this matter, but with this case study, at least a starting point for further discussion is provided.

## Electronic Supplementary Material

Below is the link to the electronic supplementary material.

Supplementary Material 1

## Data Availability

The data that support the findings of this study are available in the Supporting information.
