# Supplementary material for: Studies About the Effect of Halogenated Solvents on the Fluorescence Properties of 9-Aryl-Substituted Isoquinolinium Derivatives – A Case Study
Source: J Fluoresc. 2024 Apr 10;35(4):2407–14. doi: 10.1007/s10895-024-03691-z (PMC12041096; doi:10.1007/s10895-024-03691-z)
Supplement: Supplementary file 1 — Supplementary Material 1 [file 10895_2024_3691_MOESM1_ESM.pdf]

Supplementary Information

Studies on the effect of halogenated solvents on the emission properties of aryl-substituted isoquinolinium derivatives – A case study

Philipp Groß[1], Heiko Ihmels[1]

[1] Department of Chemistry and Biology, and Center of Micro- and Nanochemistry and (Bio)Technology (*Cµ*), University of Siegen, Adolf-Reichwein-Str. 2, 57068 Siegen (Germany).

Correspondence: Heiko Ihmels

E-mail: ihmels@chemie.uni-siegen.de

Materials

All reagents were commercially available and were purchased from the following companies:

- Alfa Aesar GmbH & Co KG (Haverhill, USA): chloroform.
- Merck KGaA (Darmstadt, Germany): bromoethane, dibromomethane.
- Roth (Karlsruhe, Germany): 1,1-dichloroethane.
- Thermo Fisher Scientific (Waltham, USA): acetonitrile, bromoform, dichloromethane.

All other reactants and solvents were commercially available and used without further purification.

Synthesis

The counter ion metathesis was performed with an analytical sample of **4b** in MeCN, which was treated with a solution of tetrabutylammonium bromide in MeCN. The resulting precipitate was filtered off to obtain **4b**^{Br}. El. Anal. for $C_{26}H_{22}NO_4Br$, calcd. (%): C 63.43, H 4.50, N 2.84, found (%): C 63.09, H 4.55, N 3.29.

Absorption and emission spectra

Solutions were prepared for each measurement from stock solutions of the berberine derivatives **4a–f** in MeOH (c = 1.0 mM). Aliquots of the stock solution were thoroughly evaporated under a stream of nitrogen, and the residue was redissolved in the respective solvent or solvent mixture. The absorption spectra were recorded in a range of 200–600 nm with a scan rate of 120 nm/min. The spectra were modified with a smoothing function "moving average", as implemented in the Origin software[1] or with the implemented smoothing function in the program "Scan".

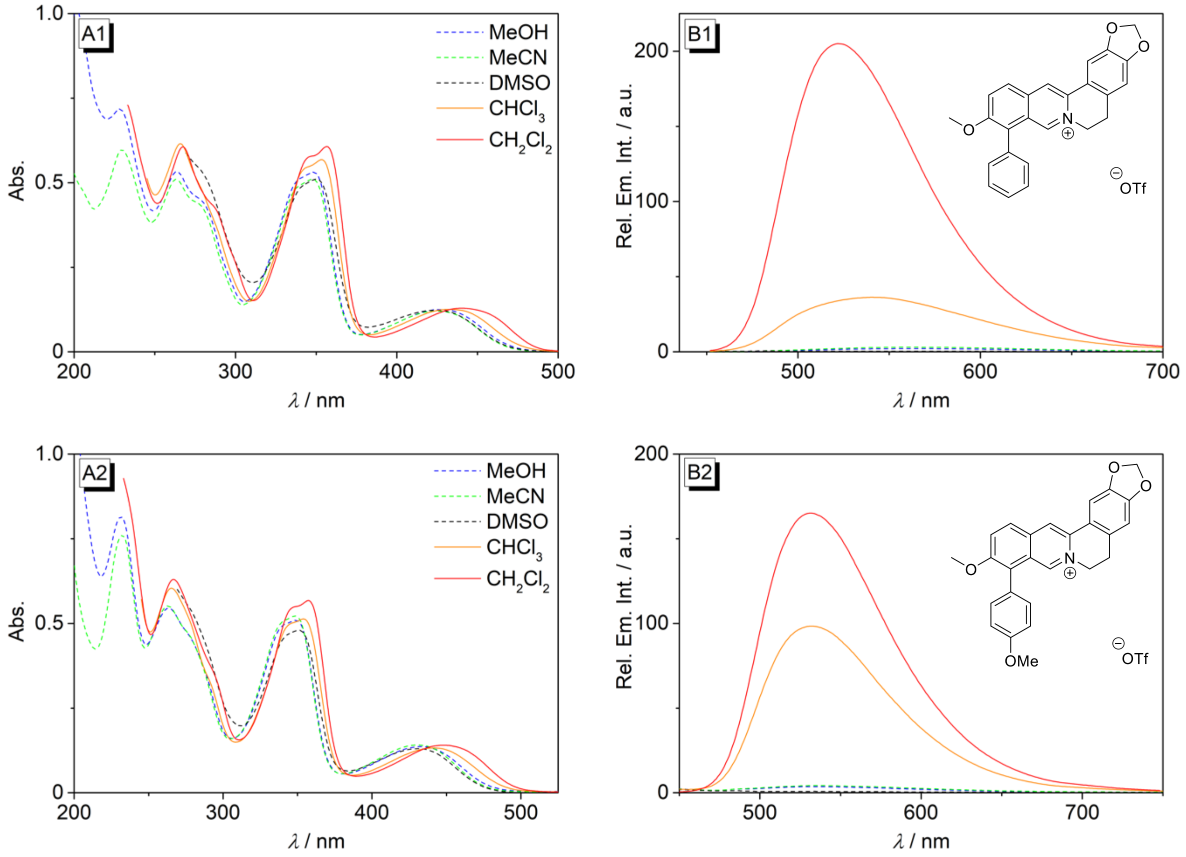

Figure S1. Absorption (A) and emission spectra (B) of **4a** (1) and **4b** (2) (c = 20 μM) in MeOH (dashed, blue), MeCN (dashed, green), DMSO (dashed, black), $CHCl_3$ (solid, orange) and CH_2Cl_2 (solid, red).

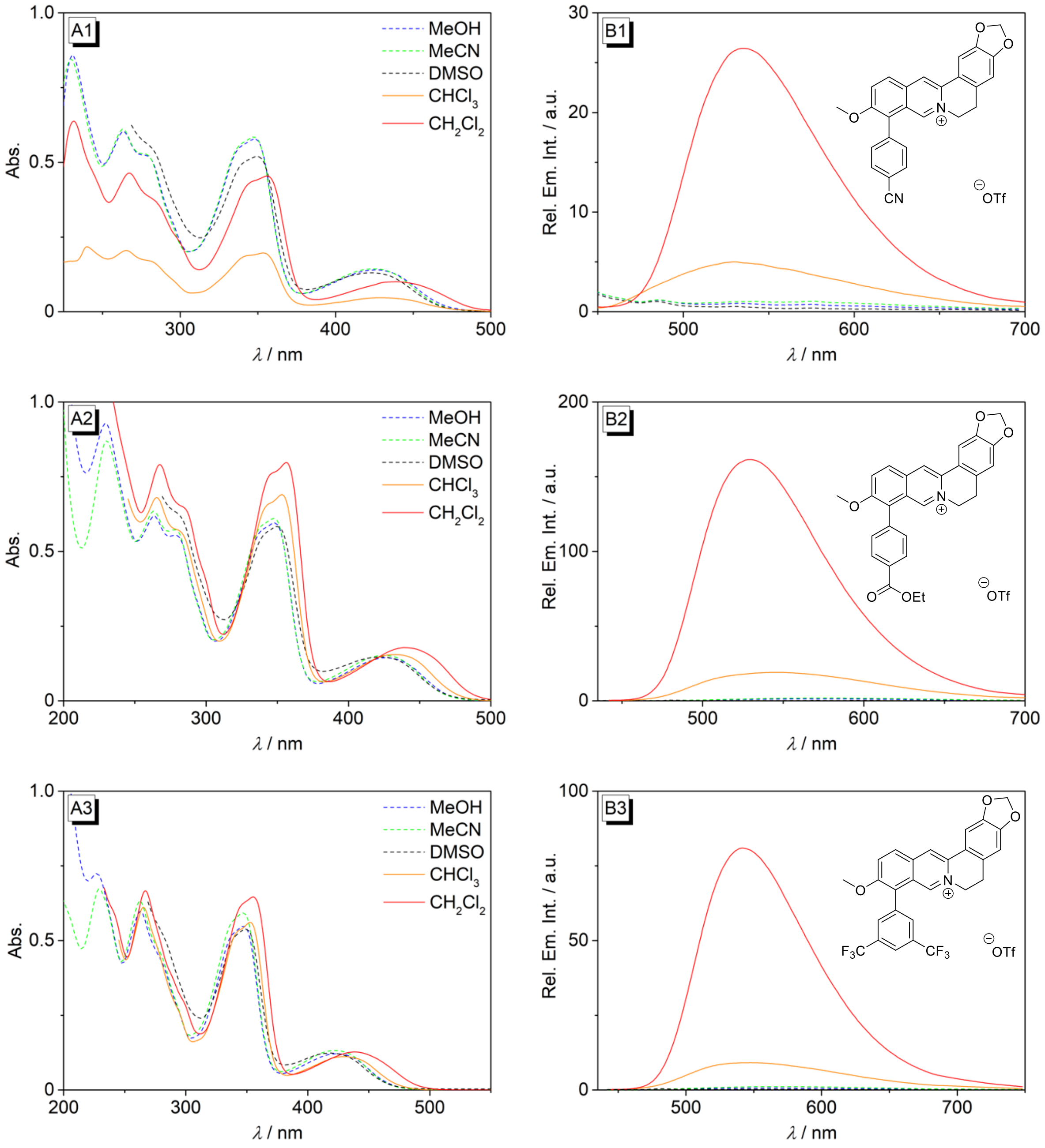

Figure S2. Absorption (A) and emission spectra (B) of **4c** (1), **4d** (2), and **4e** (3) (c = 20 μM) in MeOH (dashed, blue), MeCN (dashed, green), DMSO (dashed, black), $CHCl_3$ (solid, orange) and CH_2Cl_2 (solid, red).

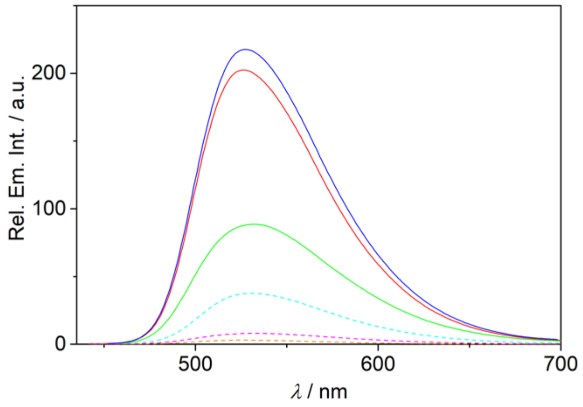

Figure S3. Fluorescence spectra of **4b** (c = 20 µM) in dichloromethane (solid, red), dibromomethane (dashed, cyan), chloroform (solid, green), bromoform (dashed, magenta), 1,1-dichloroethane (solid, blue) and bromoethane (dashed, orange).

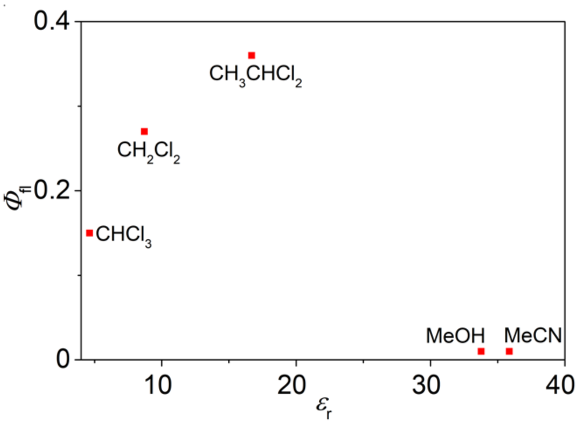

Figure S4. Plot of the fluorescence quantum yields (Φ_{fl}) of **4b** in chloroform, dichloromethane, 1,1-dichloroethane, MeCN and MeOH versus the dielectric constants (ε_r) of the respective solvent Ref. [2].

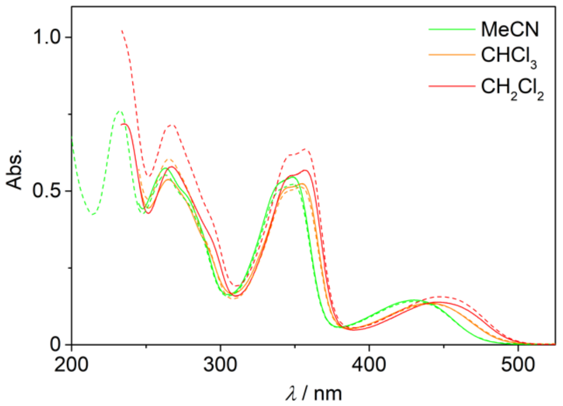

Figure S5. Absorbance spectra of **4b** (solid) and **4b**^{Br} (dashed) (c = 20 µM) in MeCN (green), chloroform (orange) and dichloromethane (red).

Theoretical quantum mechanical investigations

The optimized structure and DFT energies were calculated with the [RI BP86] or [B3LYP] method, respectively.[3] The [def2-TZVP] was used as basis set for an aqueous solution of **4b** (Figure S5).[3] Solvent properties were simulated with the implicit solvent model (CPCM).[3] The calculations were performed with ORCA Software and the input files for ORCA as well as the output files were edited with Avogadro.[4–6]

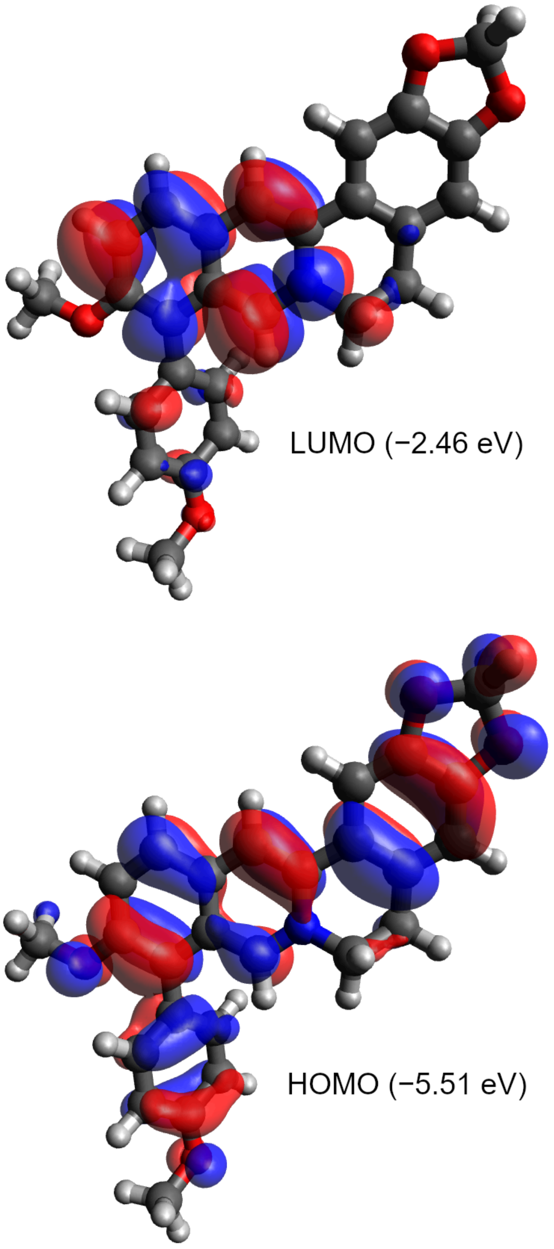

Figure S5. The frontier molecular orbitals of **4b** and their respective energies calculated by time dependent density functional theory. Highest occupied molecular orbital (HOMO, bottom) and lowest unoccupied molecular orbital (LUMO, top). Positive lobes are depicted in red and negative lobes are depicted in blue.

1. Origin Lab Cooperation, *Origin 8.5.1*, Northhampton, MA, USA, **2011**.

2. C. Wohlfarth (Ed.) *Static Dieletric Constants of Pure Liquids and Binary Liquid Mixtures*. *Supplement to IV/6*, Springer, Berlin, **2008**.

3. Foresman J. B.; Frisch A., *Exploring Chemistry with Electronic Structure Methods*, Gaussian Inc., Wallingford, CT USA, **1996**.

4. Neese F. *WIREs Comput. Mol. Sci.* **2012**, *2*, 73–78.

5. Hanwell M. D.; Curtis D. E.; Lonie D. C.; Vandermeersch T.; Zurek E.; Hutchison G. R. *J. Chemformatics* **2012**, *4*, 17–33.

6. Neese F. *WIREs Comput. Mol. Sci.* **2022**, 12.
